# PlantReg: the reconstruction of links between transcription factor regulatory networks and biological processes under their control

**DOI:** 10.18699/vjgb-24-102

**Published:** 2024-12

**Authors:** V.V. Lavrekha, N.A. Omelyanchuk, A.G. Bogomolov, E.V. Zemlyanskaya

**Affiliations:** Institute of Cytology and Genetics of the Siberian Branch of the Russian Academy of Sciences, Novosibirsk, Russia Novosibirsk State University, Novosibirsk, Russia; Institute of Cytology and Genetics of the Siberian Branch of the Russian Academy of Sciences, Novosibirsk, Russia; Institute of Cytology and Genetics of the Siberian Branch of the Russian Academy of Sciences, Novosibirsk, Russia; Institute of Cytology and Genetics of the Siberian Branch of the Russian Academy of Sciences, Novosibirsk, Russia Novosibirsk State University, Novosibirsk, Russia

**Keywords:** gene ontology, biological processes, gene regulatory networks, Arabidopsis thaliana, генная онтология, биологические процессы, регуляторные генные сети, Arabidopsis thaliana

## Abstract

The description of the path from a gene to a trait, as the main task of many areas in biology, is currently being equipped with new methods affecting not only experimental techniques, but also analysis of the results. The pleiotropic effect of a gene is due to its participation in numerous biological processes involved in different traits. A widespread use of genome-wide sequencing of transcripts and transcription factor (TF) binding regions has made the following tasks relevant: unveiling pleiotropic effects of TFs based on the functions of their target genes; compiling the lists of TFs that regulate biological processes of interest; and describing the ways of TF functioning (their primary and secondary targets, higher order targets, TF interactions in the process under study). We have previously developed a method for the reconstruction of TF regulatory networks and proposed an approach that allows identifying which biological processes are controlled by these networks and how this control is exerted. In this paper, we have implemented the approach as PlantReg, a program available as a web service. The paper describes how the program works. The input consists of a list of genes and a list of TFs – known or putative transcriptional regulators of these genes. As an output, the program provides a list of biological processes enriched for these genes, as well as information about by which TFs and through which genes these processes are controlled. We illustrated the use of PlantReg deciphering transcriptional regulation of processes initiated at the early salt stress response in Arabidopsis thaliana L. With PlantReg, we identified biological processes stimulated by the stress, and specific sets of TFs that activate each process. With one of these processes (response to abscisic acid) as an example, we showed that salt stress mainly affects abscisic acid signaling and identified key TFs in this regulation. Thus, PlantReg is a convenient tool for generating hypotheses about the molecular mechanisms that control plant traits.

## Abstract (ru)

Описание пути от гена к признаку как основная задача многих отраслей биологии в настоящее время оснащается новыми методами не только в технике экспериментов, но и в системном анализе их результатов. Плейотропный эффект гена осуществляется за счет его участия во многих биологических процессах, вовлеченных в разные признаки. Широкое распространение полногеномного секвенирования транскриптов и районов связывания транскрипционных факторов (ТФ) сделало актуальными задачи установления плейотропных эффектов ТФ за счет знаний о функциях их мишеней, составление списков ТФ, регулирующих интересующие исследователя биологические процессы, описание путей их действия (первичные и вторичные мишени, мишени следующих порядков, взаимодействие между ТФ в исследуемом процессе). Ранее мы разработали метод реконструкции регуляторных сетей ТФ и предложили подход, позволяющий выявлять, какие биологические процессы и каким образом эти сети регулируют. В данной работе мы реализовали этот подход в виде программы PlantReg, доступной пользователям через веб-интерфейс. В статье описан принцип работы программы. На вход подаются список генов и список ТФ – известных или предполагаемых регуляторов транскрипции этих генов. На выходе программа выдает список биологических процессов, которые обогащены в этих генах, а также информацию о том, какими ТФ и через какие гены эти процессы регулируются. Работа PlantReg проиллюстрирована на примере исследования регуляции процессов, инициируемых на начальных этапах ответа на солевой стресс у Arabidopsis thaliana L. С помощью программы PlantReg нами выявлены биологические процессы, стимулируемые в раннем ответе на солевой стресс, и специфический набор ТФ, активирующих каждый из этих процессов. На примере одного из таких процессов – ответа на фитогормон абсцизовую кислоту – мы показали, что солевой стресс активирует в основном сигнальный путь этого гормона, и выделили ключевые ТФ в этой регуляции. Таким образом, программа PlantReg – удобный инструмент для создания гипотез о молекулярных механизмах регуляции признаков растений

## Introduction

The efficient development of transcriptome sequencing methods has opened up wide opportunities not only to study changes in gene expression at the level of transcription, but also to track the regulation of these changes by transcription factors (TFs) and their impact on biological processes (Chen J.W. et al., 2023). In this regard, methods for compilation of TF lists based on the presence of their binding sites in the promoters of differentially expressed genes (DEGs) and methods for gene ontology (GO) terms enrichment analysis of gene lists (i. e., their functional annotation) are now widely used. Nevertheless, identification of the relationship between the outputs of these methods (i. e., determination of TFs that affect specific biological processes, their stages influenced by these TFs, common and specific TFs among the processes) remains a poorly worked out part in the analysis of transcriptomic data. The development of computer programs for this purpose will make this analysis more systematic and build a connection between alterations in gene expression and changes in biological processes.

If TFs regulate each other at the transcription level, their interactions are often represented as graphs – transcription factor regulatory networks (TFRNs), which can be reconstructed using various methods (Hecker et al., 2023). TFRNs allow establishing hierarchy in their architecture and identifying hubs – TFs that are most connected to other TFs. Altering the expression of genes encoding hubs is likely to change the functioning of the entire TFRN, and consequently affects downstream biological processes (He, Zhang, 2006).

We have previously developed a methodology and a software for reconstruction of TFRNs. We have also proposed a bioinformatics approach to identify biological processes under control of TFRNs and regulatory links between TFRN components and the processes (Omelyanchuk et al., 2024). It is based on the following steps. The first step is compilation of a list of TFs enriched in DEG promoters. The TF list is then used for TFRN reconstruction. The second step is functional annotation of the DEG list, after which within every biological process potential regulators of each of its DEGs are extracted from the TF list composed at the first step. After this, the genes are arranged in the order in which they function during a biological process, and the TFs that control the individual stages of this process can be identified. The use of this approach was illustrated in (Omelyanchuk et al., 2024) with the examples of auxin regulation of chlorophyll and lignin biosynthesis, abscisic acid signaling, and ribosome biogenesis

In this work, we implemented this approach as a PlantReg program, available to users via a web interface (https:// plamorph.sysbio.ru/fannotf/). We used PlantReg to investigate the regulation of processes during an early salt stress response in Arabidopsis thaliana L., using transcriptomic data from (Wu et al., 2021a). With PlantReg, we found that processes involved in the early reaction to salt stress and coordinated by all TFs within the TFRN include responses to heat, red and far-red light, and salicylic acid. The largest number of processes (programmed cell death, leaf senescence, and responses to blue light, hypoxia, reactive oxygen species, dehydration, abscisic acid, and jasmonic acid) are regulated by at least 70 % of TFs from the TFRN. In the control of the endoplasmic reticulum (ER) unfolded protein response, biosynthesis of indole-containing compounds and S-glucosides, as well as water transport, less than 50 % of the TFRN is involved.

Next, we examined the PlantReg results on the regulation of the abscisic acid (ABA) response during early salt stress in more detail and found that this regulation is primarily mediated through the control of ABA signaling, and its last stage, activation of the master TFs, is modulated most strongly. Both TFRN hubs (WRKY8 and DEAR2) are involved in this activation, and DEAR2 also controls ABA reception. Thus, the PlantReg program is an effective tool for analyzing data on differential gene expression in transcriptomes and creating hypotheses about the molecular mechanisms operating in regulation of biological processes

## Materials and methods

**Fig. 1.**
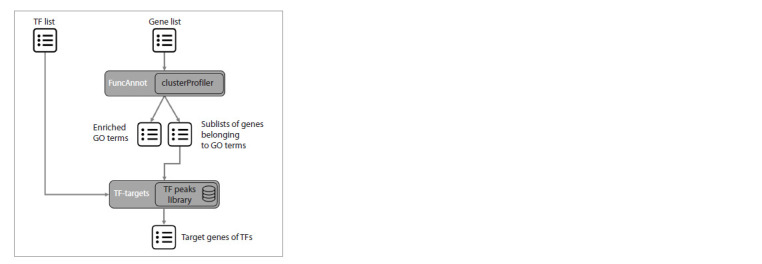
The PlantReg workflow

**PlantReg implementation. **PlantReg workflow is shown in Figure 1. The program takes a list of genes (in this work, we focus on DEG lists) and a list of TFs that are known or putative transcriptional regulators of these genes as input. The FuncAnnot function performs functional annotation of the gene list using the clusterProfiler R package (Yu et al., 2012; Wu et al., 2021b). The result is a file containing information about the GO terms enriched in the DEG list, as well as sublists of genes from the input annotated with the enriched GO terms. The next step is the search for the overlaps between the binding peaks of the input TFs and 5′ regulatory regions of genes from the sublists. For this purpose, the TF-targets function, which we developed earlier as part of the CisCross-FindTFnet program (Omelyanchuk et al., 2024), is applied. As output, the user receives a file containing enriched GO terms and their associated DEGs, evidence codes, and TFs, the binding peaks of which are mapped to the 5ʹ regulatory regions of DEGs associated with the enriched GO terms

The core of the PlantReg software is implemented in Perl and recruits the clusterProfiler R package. PlantReg is accessible through a web interface (https://plamorph.sysbio.ru/ fannotf/). In the web version of PlantReg, two collections of TF binding profiles are available for identifying target genes of TFs. The first collection (GTRD-MACS2) includes 306 sets of ChIP-seq peaks for 131 A. thaliana TFs downloaded in BED format from the GTRD database (https://gtrd.biouml.org/#!) (Kolmykov et al., 2021). The second collection (CisCross- MACS2) was obtained by large-scale profiling of A. thaliana TF binding sites using DAP-seq (O’Malley et al., 2016) and represents the result of re-processing of raw data from the original study (Lavrekha et al., 2022). This collection contains 608 peak sets for 404 TFs of A. thaliana. The ARAPORT11 annotation of A. thaliana genome (https://bar.utoronto.ca/ thalemine/begin.do) is used to identify 5ʹ regulatory regions of genes (500, 1,000, 1,500, 2,000, or 2,500 bp upstream of the transcription start) in the PlantReg web version.

**Reconstruction of the TFRN for early response to salt stress.** To reconstruct the TFRN for early response to salt stress, we used publicly available RNA-seq data for sevenday- old A. thaliana seedlings (ecotype Col-0) grown in the light, before and after salt treatment (100 mM NaCl) for 1 h (Wu et al., 2021a). To extract DEGs, we set the FDR threshold at 0.05; among them, we distinguished upregulated and downregulated DEGs (uDEGs and dDEGs, respectively). The TFRN was reconstructed using the CisCross-FindTFnet program (Omelyanchuk et al., 2024) with the following parameters. For mapping of TF binding regions, we used the CisCross-MACS2 collection of peaks, and set the length of the 5ʹ regulatory regions to 1,000 bp. The positions of transcription start sites were determined according to the ARAPORT11 A. thaliana genome annotation. In TF binding peak enrichment analysis of 5′ regulatory regions of uDEGs and dDEG, we controlled FDR at 0.001 using the Benjamini– Hochberg method. To reconstruct “TF-regulator–TF-target” pairs within the TFRN, we used the peak sets corresponding to the binding of TFs to the native leaf genomic DNA possessing methylation marks.

**Reconstruction of the links between the TFRN for early response to salt stress and the biological processes it controls.** Using PlantReg, we reconstructed the links between the TFRN for early response to salt stress and downstream biological processes. As input, we used a list of TFs from the TFRN, as well as a list of DEGs responding to salt treatment (uDEGs and dDEGs were analyzed separately). The length of the 5ʹ regulatory regions was set to 1,000 bp, and the CisCross-MACS2 collection was used to map TF binding peaks. For further analysis and interpretation, we only used “TF-regulator–Target gene” pairs reconstructed based on DAP-seq TF binding profiles captured in leaf genomic DNA possessing methylation marks.

## Results and discussion

Biological interpretation of PlantReg output data

The PlantReg program is designed to reconstruct molecular mechanisms operating in genetic regulation of traits. To get started, the user needs to have a list of known or putative regulators of differential gene expression. PlantReg performs a functional annotation for the list of DEGs, then searches for potential targets of TFs among DEGs associated with enriched biological processes. The mapping of TF binding peaks in the 5ʹ regulatory regions of genes is performed using a representative collection of whole-genome TF binding profiles for the species being studied. In the web version, two collections of TF binding profiles for A. thaliana, from ChIP-seq or DAPseq data, are available. The user can choose one of them. The program outputs the relationships between DEGs, the upstream TFs, and the enriched GO terms

**Fig. 2.**
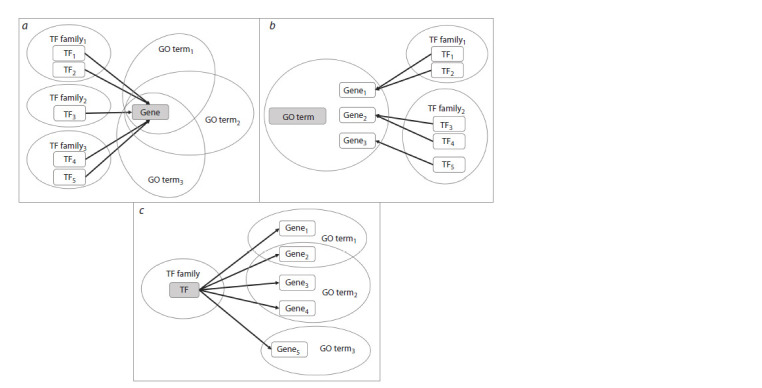
The PlantReg output representations Panels a, b and c correspond to output blocks 1, 2 and 3. The central output element is highlighted in gray.

For convenient biological interpretation and subsequent analysis, PlantReg output is organized in five blocks. The first four blocks offer four alternative representations of the same results. So, block (1) characterizes genes. It contains a sublist of DEGs annotated with the enriched GO terms, the list of potential TFs (with an indication of TF family) and the number of TFs for each DEG (Fig. 2a). Each DEG is also characterized with the total number and the list of enriched GO terms (with an indication of the evidence code), which facilitates identification of DEGs involved in a wide range of biological processes as well as DEGs specific to particular processes

Biological processes are the focus of block (2). In this block, for each enriched GO term, a sublist of associated DEGs with the evidence codes is created, as well as a sublist of TFs potentially regulating the expression of these DEGs with an indication of TF family (Fig. 2b). This output block allows reconstructing the mechanism of genetic regulation for each biological process

Block (3) characterizes transcriptional regulators of differential gene expression. It contains a list of TFs, for which the target genes associated with enriched GO terms were found among DEGs (Fig. 2c). This output representation is useful for planning the experiments to verify the predicted mechanisms for genetic regulation of biological processes.

Block (3) characterizes transcriptional regulators of differential gene expression. It contains a list of TFs, for which the target genes associated with enriched GO terms were found among DEGs (Fig. 2c). This output representation is useful for planning the experiments to verify the predicted mechanisms for genetic regulation of biological processes.

Block (4) holds a table where each row contains one DEG, one of the TFs potentially regulating its expression, its family, and one of the GO terms with the evidence codes. This output can be used for further analysis with software tools

The auxiliary block (5) accommodates the results of functional annotation of DEGs by clusterProfiler with the significance of GO term enrichment.

Functional annotation of the TFRN for early response to salt stress in A. thaliana

We used the PlantReg program to investigate the mechanisms that regulate the response to salt stress in the model plant species A. thaliana. A list of DEGs that respond to high salt concentration was extracted from publicly available transcriptome data (Wu et al., 2021a). In order to generate a list of potential TF regulators for these genes, we used the previously developed CisCross-FindTFnet tool. Based on the combined analysis of DEGs and TF binding profiles, this tool identifies potential TF regulators of DEGs, classifies them by regulation type, determines the relationships between them and reconstructs a TFRN (Omelyanchuk et al., 2024).

TF regulation types are distinguished based on a set of rules and correspond to the following properties of the regulators. US (upregulated suppressor) is a suppressor induced by the stimulus (in our case, high salt concentration). It suppresses the expression of target genes that were active before the stimulus application. UA (upregulated activator) is induced by the stimulus and activates expression of its target genes. DA (downregulated activator) and DS (downregulated suppressor) are active in the absence of the stimulus. The application of the stimulus inhibits DA expression and, consequently, expression of its target genes. DS suppresses activity of its target genes in the absence of the stimulus; under the stimulus exposure, DS expression is reduced and the activity of its targets is unblocked.

**Fig. 3.**
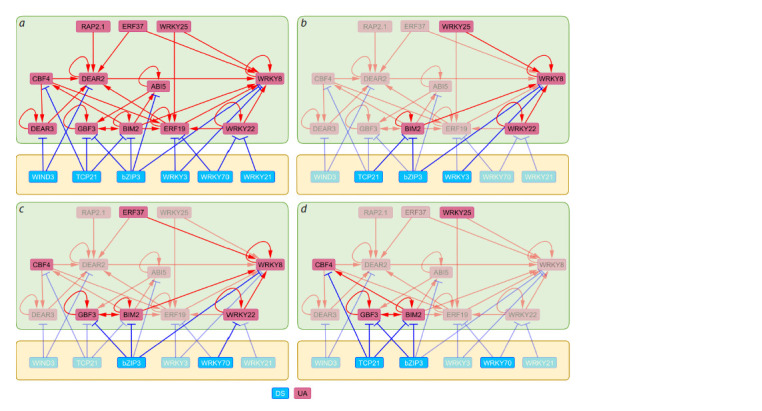
The reconstructed TFRN for the early salt stress response in A. thaliana (a) and its participation in the regulation of processes that compose the salt stress response: ER unfolded protein response (b), biosynthesis of indole-containing compounds (c) and S-glycosides (d). The nodes of the graphs correspond to transcription factors. TF1 and TF2 are connected by an edge directed from TF1 (regulator) to TF2 (target) if the TF1 binding peak is mapped in the 5’ regulatory region of the TF2-encoding gene. The green block highlights the group of TFs (UAs) that are activated and activate their targets in response to salt stress. The yellow block highlights the group of TFs (DSes) that repress genes normally and are themselves repressed by salt stress, which results in passive activation of the DS targets. The nodes and edges involved in the regulation of the process are highlighted in panels b–d. DSes and UAs denote downregulated suppressors and upregulated activators according to (Omelyanchuk et al., 2024).

The structure of the early salt stress response TFRN reconstructed with the CisCross-FindTFnet program is shown in Figure 3a, and consists only of TFs, the binding sites of which were enriched in uDEGs, i. e., the response to salt stress begins with transcription activation, and TFs in the TFRN are related only to the DS and UA types, i. e., gene activation occurs passively due to stress-induced downregulation of the suppressor (DS) or actively due to stimulation of the activator (UA). Among UA-encoding genes, increased activity under salt stress was previously experimentally shown for CBF4/ DREB1D (Sakuma et al., 2002), ERF37/DREB A-4 (Hossain et al., 2016), RAP2.1/DEAR6 (Ghorbani et al., 2019), WRKY25 (Jiang, Deyholos, 2009), ABI5 (Yuan et al., 2011),GBF3 (Zhang L. et al., 2012, 2017) and WRKY8 (Hu et al., 2013). Wherein, ABI5 (Yuan et al., 2011), GBF3 (Zhang L. et al., 2012, 2017), and WRKY8 (Hu et al., 2013) play a key role in response to salt stress.

For DSes we identified, it was previously demonstrated that inactivation of WRKY70 increased plant tolerance to salt stress (Li J. et al., 2013), and bZIP3 expression was inhibited by salt stress (Liu Y. et al., 2013). Notably, longer salt stress (4 h) activated WRKY3 (Li P. et al., 2021). Thus, the composition of our reconstructed TFRN is in good accordance with the published data. At the same time, only four TFs out of 18 (22 %) have been previously identified as the key players in salt stress, and only 10 (56 %) have been described to respond to salt stress, i. e., the TFRN contains new potential regulators of this process.

Functional annotation of DEGs showed that the early response to salt stress is accompanied by the ER unfolded protein response, as well as activation of the following processes: programmed cell death, leaf senescence, water transport, biosynthesis of indole-containing compounds and S-glycosides, response to heat, red and far-red light, abscisic, salicylic and jasmonic acids, blue light, hypoxia, reactive oxygen species, and dehydration. A link between the response to salt stress and heat has been shown previously, as heat shock proteins enhance resistance to salt stress and, conversely, overexpression of salt stress proteins provides resistance to heat stress (Azameti et al., 2024; Chaffai et al., 2024; Chang et al., 2024). The relationship of salt stress response to leaf senescence, hypoxia, water transport, responses to blue, red, and far-red light, reactive oxygen species, dehydration, abscisic acid, salicylic acid, and jasmonic acid has also been demonstrated in experiments (Serraj et al., 1994; Szepesi et al., 2009; Khan et al., 2012; Kumar et al., 2014; Joseph, Jini, 2010; Sharma et al., 2022; Kesawat et al., 2023; Lu, Fricke, 2023; Tan et al., 2023; Peng et al., 2024).

Salt stress leads to disruption of protein folding in the endoplasmic reticulum (so-called endoplasmic reticulum stress), and the response to this is optimization of protein folding, resulting in a decrease in unfolded proteins (Liu et al., 2007; Wang et al., 2011). There is evidence for the involvement of biosynthesis of an indole-containing compound such as melatonin in the response to salt stress (Qi et al., 2020; Shamloo- Dashtpagerdi et al., 2022). Enrichment of salt stress response genes with the gene ontology term “S-glycoside metabolism” has been detected previously (Rodriquez et al., 2021).

We found that all TFs in the TFRN are involved in the regulation of the response to heat, red and far-red light, and salicylic acid. The remaining biological processes fell into two groups: those controlled by at least 70 % of the network TFs and those controlled by less than 50 % of the network TFs. The first group included programmed cell death, leaf senescence, and responses to blue light, hypoxia, reactive oxygen species, dehydration, abscisic acid, and jasmonic acid. The second group comprised the ER unfolded protein response (Fig. 3b), biosynthesis of indole-containing compounds (Fig. 3c) and S-glycosides (Fig. 3d), and water transport (the latter was regulated by only three TFs: BIM2, bZIP3, and WIND3). Thus, using PlantReg, we have shown that the response to salt stress is composed of both processes regulated by the entire TF network and processes controlled by distinct parts of this network.

Among the TFs we have identified as controlling the ER unfolded protein response, only WRKY70 has been shown as a regulator of this process to date (Wang L.Y. et al., 2023), and bZIP3 has been indicated as a possible candidate for this role (Ko et al., 2023).

Glucosinolates, the most diverse and studied group of S- glycosides, are the secondary metabolites of Brassicaceae involved in plant defense (Halkier, Gershenzon, 2006). Currently, they are intensively studied due to their therapeutic and preventive properties against cancer, cardiovascular or neurological diseases. Glucosinolates are categorized into three groups depending on the amino acids from which they are derived: aliphatic glucosinolates (methionine, alanine, leucine, isoleucine, and valine), aromatic glucosinolates (phenylalanine and tyrosine), and indole glucosinolates (tryptophan). For at least three out of seven TFs that we found to control glucosinolate biosynthesis, this function was previously known. CBF4 triggers the synthesis of aliphatic glucosinolates, which also increases salt stress tolerance (Defoort et al., 2018), while WRKY70 suppresses indole-3-ylmethyl glucosinolate biosynthesis (Li J. et al., 2006). GBF3 expression is significantly reduced in mutants for the SUR2/CYP83B1 gene that controls the metabolic switch between auxin and indole glucosinolate biosynthesis (Morant et al., 2010).

Regulation of abscisic acid signaling pathway under salt stress in A. thaliana

In addition to determining the composition of TFs that control specific processes, PlantReg allows determination of TFs that regulate the activity of individual genes in these processes. The latter provides an opportunity to identify modulators of gene expression consistently at each stage of the process. In this paper, we demonstrate this on the example of reconstructing the mechanism for transcriptional regulation of ABA response under salt stress. According to PlantReg results, all TFs within the salt stress response TFRN except for WRKY21 control ABA response. This regulation starts with the control of ABA level in the cell

**Fig. 4.**
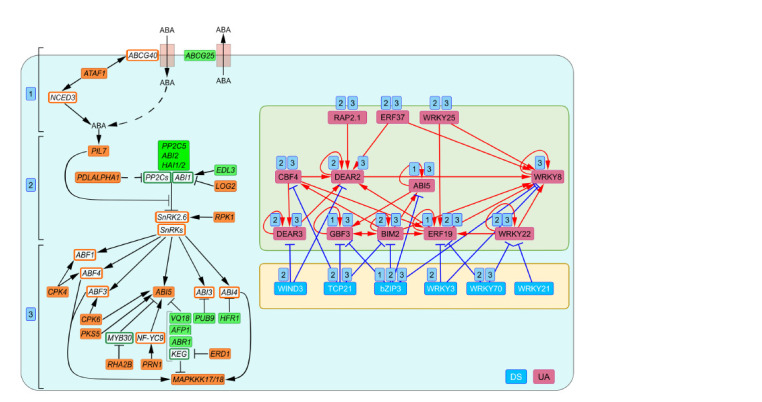
Transcriptional regulation of ABA level and signaling under early salt stress. Green and orange rectangles denote uDEGs that encode repressors and activators of the ABA level and signaling pathway, respectively, and are potential TFRN targets. White rectangles in green and orange frames correspond to repressors and activators of ABA level and signaling that are not potential TFRN targets. Numbers in blue rectangles denote the following stages: 1 – control of ABA level; 2 – ABA perception by receptors; 3 – activation of master TFs of ABA response. Abbreviations for the names of ABA transport, biosynthesis and signaling genes: ATP-BINDING CASETTE G25/40 (ABCG25/40), PYR1 LIKE 7 (PYL7), PROTEIN PHOSPHATASES TYPE 2C (PP2Cs), ABA INSENSITIVE1/2/3/4/5 (ABI1/2/3/4/5), SNF1-RELATED PROTEIN KINASE (SnRKs), ABSCISIC ACID RESPONSIVE ELEMENT-BINDING FACTOR1/3/4 (ABF1/3/4), CALCIUM-DEPENDENT PROTEIN KINASE 4/6 (CPK4/6), ABI FIVE BINDING PROTEIN 1 (AFP1), KEEP ON GOING (KEG), ENHANCED DISEASE RESISTANCE 1 (EDR1), NUCLEAR FACTOR Y9 (NF-YC9), PLANT U-BOX/ARM-REPEAT (ATPUB-ARM) E3 LIGASE 9 (PUB9), ABA REPRESSOR 1 (ABR1), VQ PROTEIN 18 (VQ18), HIGHLY ABA-INDUCED PP2C GENE 1/2 (HAI1/2), ARABIDOPSIS THALIANA ACTIVATING FACTOR1 (ATAF1), EID1-LIKE 3 (EDL3), LONG HYPOCOTYL IN FAR-RED 1 (HFR1), LOSS OF GDU2 (LOG2), MITOGEN-ACTIVATED PROTEIN KINASE KINASE KINASE 17/18 (MAPKKK17/18), PHOSPHOLIPASE D ALPHA 1 (PLDALPHA1), PIRIN 1 (PRN1), RING-H2 FINGER PROTEIN 2B (RHA2B), RECEPTOR-LIKE PROTEIN KINASE 1 (RPK1), CALCINEURIN B-LIKE PROTEIN-INTERACTING PROTEIN KINASEs/SOS2-LIKE PROTEIN KINASE (PKS5), MYB DOMAIN PROTEIN 30 (MYB30), NINE-CIS-EPOXYCAROTENOID DIOXYGENASE 3 (NCED3).

At this stage (stage 1 in Figure 4), the targets of the TFRN include the ABCG25 and ATAF1 genes encoding, respectively, the ABA exporter from the cell (Park et al., 2016) and the TF that activates both the ABA biosynthesis gene NCED3 (Jensen et al., 2013) and the ABA importer gene ABCG40 (Kang et al., 2015).

In the next stage (stage 2 in Figure 4), ABA binds to and activates the PYRABACTIN RESISTANCE1/PYR1 LIKE/ REGULATORY COMPONENTS OF ABA RECEPTORS (PYR/PYL/RCAR) group of receptors (Fidler et al, 2022), among which the salt stress response TFRN controls PYL7. It is the most tightly TFRN-controlled gene in ABA signaling, since its expression is managed by half of the TFRN TFs (9 of 18). Under normal conditions, PYL7 activity is suppressed by bZIP3 and WIND3. Whereas bZIP3 inhibits the activity of 11 ABA signaling genes in addition to PYL7, WIND3 is a specific suppressor of PYL7. Salt stress activates PYL7 through seven TFs that form a regulatory loop with DEAR2 being a hub, directly activated by five TFs (CBF4, DEAR3, ERF19, ERF37, RAP2.1), while the sixth TF (WRKY22) stimulates it through ERF19.

In ABA signaling, PYR/PYL/RCAR receptors inhibit PP2C phosphatase activity, thereby preventing dephosphorylation of SnRK2 kinases (Fidler et al., 2022). Here, the direct TFRN targets are genes encoding the following: PP2C phosphatases PP2C5, ABI2 and HAI/2, as well as the SNRK2.6 activator RPK1 (Shang et al., 2020), PP2C phosphatase regulators EDL3 (Koops et al, 2011), LOG2 (Pan W. et al., 2020), and phospholipase PLDALPHA1, the product of which (phosphatidic acid) inhibits the activity of some PP2C phosphatases (Ndathe, Kato, 2024).

The third stage of ABA signal transduction (stage 3 in Figure 4) begins with the activation of ABA response master TFs by SnRK2 kinases. Notably, one of them, ABI5, is also represented in the TFRN. Except for ABI5 and MAPKKK17/18 (initiators of the MAPK cascade) (Zhou M. et al., 2021; Zhao et al., 2023), all other TFRN targets at this stage represent regulators of ABA response master TFs. These include genes encoding kinases CPK4/6, PKS5, EDR1 (Zhu et al., 2007; Wawrzynska et al., 2008; Zhou X. et al., 2015; Zhang H. et al., 2020), transcription factors ABR1 (Sanyal, Pandey, 2024) and HFR1 (Wang Z. et al., 2024), transcriptional regulators VQ18 (Pan J. et al., 2018) and PRN1 (Warpeha et al., 2007), components of the protein degradation complexes PUB9 (Samuel et al., 2008), AFP1 (Lopez-Molina et al., 2003), and RHA2B (Li H. et al., 2011).

Interestingly, within the TFRN, half of DSes and all UAs are involved in the control of the third step of ABA signaling. Both TFRN hubs, DEAR2 and WRKY8, have targets at this stage. Moreover, while DEAR2 has targets at stage 2 as well, WRKY8 is specific for stage 3. WRKY8 and DEAR2 enhance transcription of seven and six activators, respectively. During viral infection, WRKY8 controls ABA signaling as an infection-suppressed activator of ABI4 (Chen L. et al., 2013). We showed that under salt stress, WRKY8 controls ABA signaling by upregulating CPK6. CPK6 kinase stimulates ABF4 and ABI5 through their phosphorylation (Zhang H. et al., 2020). This suggests that the same TF may have different targets in ABA signaling under various stresses.

Thus, PlantReg demonstrated that within ABA response, the targets of the salt stress response TFRN belong to the genes involved in ABA signaling, in which the most stringent control occurs at the regulation of the master TFs, ABF1/3/4 and ABI3/4/5. Moreover, ABI5, one of the master TFs in ABA signaling, is also one of the TFs within the TFRN of the salt stress response, where its activity is suppressed by bZIP3 before stress and stimulated by BIM2 during stress. ABI5 itself activates GBF3, which, like BIM2, is repressed by bZIP3 before stress. At the same time, GBF3 and BIM2 mutually activate each other. Thus, BIM2, bZIP3, GBF3, and ABI5 form a clear regulatory circuit in our reconstructed TFRN (Fig. 3a, 4).

Interestingly, in the ABA response gene network in (Aerts et al., 2024), the TFs that make up this regulatory loop (BIM2, bZIP3, GBF3, and ABI5) belong to the group of the earliest regulators and share a large number of common targets, i. e. control the same genes. In addition to BIM2, bZIP3, GBF3, and ABI5, our reconstructed TFRN for the salt stress response overlaps with the abscisic acid response gene network from (Aerts et al., 2024) for three other TFs: CBF4, DEAR2, and WRKY3. We identified DEAR2 as a TFRN hub. Moreover, CBF4, DEAR2, and WRKY3 are components of the network connecting its central activating regulatory circuit (BIM2, GBF3, and ABI5) to the second TFRN hub, WRKY8.

WRKY3, along with bZIP3, suppresses WRKY8 before stress (Fig. 3a). Under stress conditions, sequential activation of BIM2, CBF4, DEAR2, and WRKY8 occurs. Thus, comparison of the PlantReg results with the abscisic acid response gene network (Aerts et al., 2024) identified TFs that are the key regulators of ABA response. The remaining TFs, RAP2.1, ERF19/37, DEAR3, TCP21, WRKY8/22/25/70, are possibly involved in the control of ABA signaling only under salt stress

## Conclusion

The PlantReg program has shown its efficiency in systematic analysis of the results of whole-genome experiments on differential gene expression. It allows, along with functional annotation of DEGs, identifying TF targets among them and, based on this, identifying TFs regulating certain biological processes. Combination of PlantReg results with those of programs that reconstruct TFRNs (e. g., CisCross-FindTFnet) allows subdividing a TFRN into subnetworks, which control distinct processes, to identify key TFs in these processes and even at their certain stages. The approaches and methods developed for PlantReg implementation can be successfully used to reconstruct the mechanisms of transcriptional regulation of biological processes in various species

## Conflict of interest

The authors declare no conflict of interest.
